# Supplementary material for: Who Is Afraid of CRP? Elevated Preoperative CRP Levels Might Attenuate the Increase in Inflammatory Parameters in Response to Lung Cancer Surgery
Source: J Clin Med. 2020 Oct 18;9(10):3340. doi: 10.3390/jcm9103340 (PMC7603240; doi:10.3390/jcm9103340)
Supplement: Supplementary file 1 [file jcm-09-03340-s001.pdf]

Supplementary Materials

Who Is Afraid of CRP? Elevated Preoperative CRP Levels Might Attenuate the Increase in Inflammatory Parameters in Response to Lung Cancer Surgery

Moritz Mecki Meyer [1,†], Leon Brandenburg [1,†], Helge Hudel [2], Alisa Agné [1], Winfried Padberg [1], Ali Erdogan [3], Holger Nef [4], Anca-Laura Amati [1], Oliver Dörr [4], Biruta Witte [1,†] and Veronika Grau [1,*,†]

Figure S1. Time-course of C-reactive protein, blood leukocyte counts and body temperature in patients, who underwent pulmonary resection for the treatment of lung cancer

Figure S2. Serum lipids in patients suffering from mild coronary heart disease, who were investigated before treatment, after four weeks of treatment with atorvastatin or another four weeks of combined treatment with atorvastatin and acetylsalicylic acid

Figure S3. Cholinergic control of the release of interleukin-1β (IL-1β) by monocytes isolated from the blood of patients suffering from low-grade coronary heart disease

Figure S4. Release of lactate dehydrogenase by monocytes isolated from the blood of patients suffering from low-grade coronary heart disease

Figure S5. Inhibition of the release of monocytic interleukin-1β by nicotinic agonists acetylcholine, phosphocholine, nicotine, L-α-glycerophosphocholine, or dipalmitoyl phosphatidylcholine,

Table S1. Levels of preoperative CRP in patients grouped according to their respective tumor stages

Table S2. Subgroup analysis regarding smoking behavior

Table S3. Subgroup analysis regarding surgical access

Table S4. Subgroup analysis regarding the extent of pulmonary resection

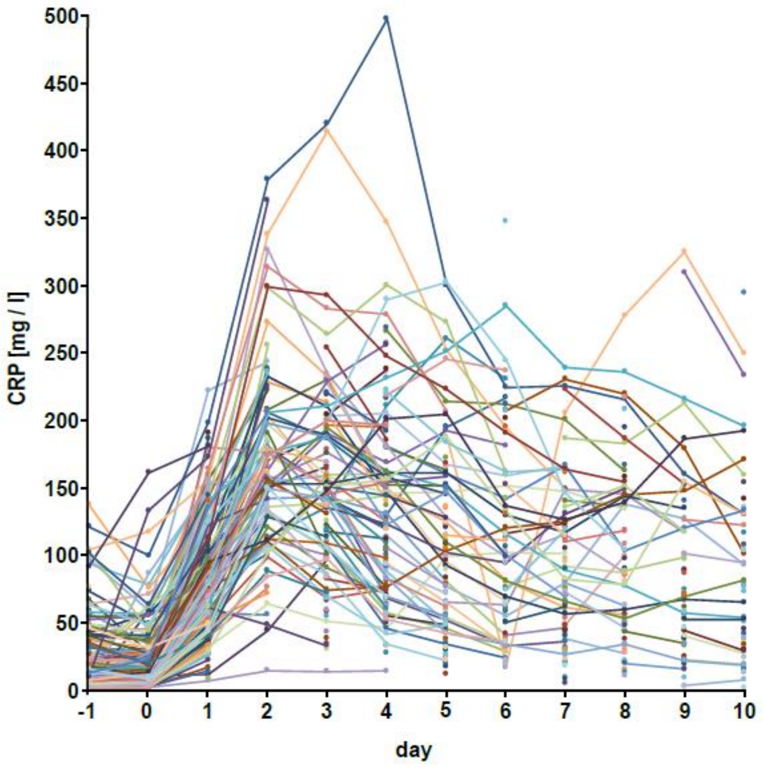

Figure S1a. Time-course of C-reactive protein (CRP) in patients, who underwent pulmonary resection for the treatment of lung cancer. Data from individual patients are symbolized by different colors, and data points belonging to the same individual are connected by lines.

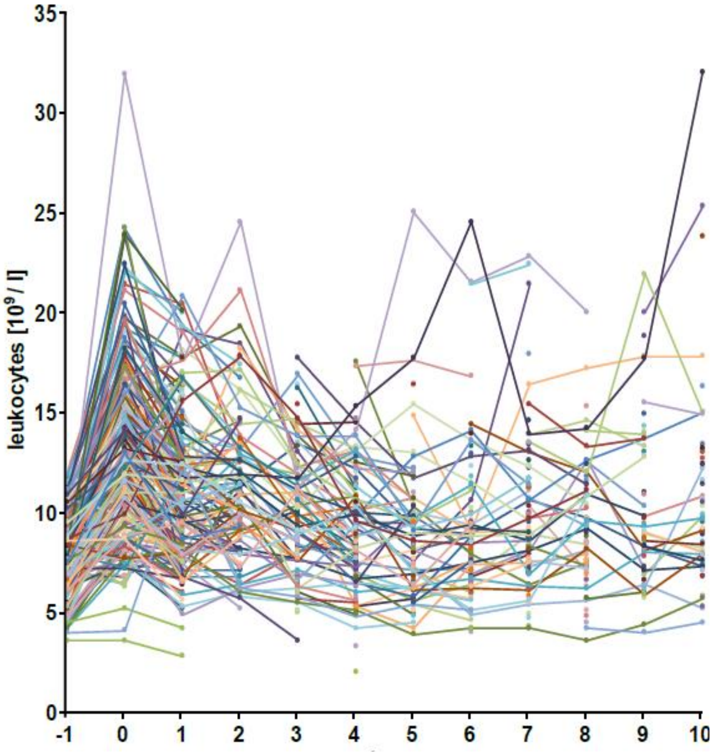

Figure S1b. Time-course of blood leukocyte counts in patients, who underwent pulmonary resection for the treatment of lung cancer. Data from individual patients are symbolized by different colors, and data points belonging to the same individual are connected by lines.

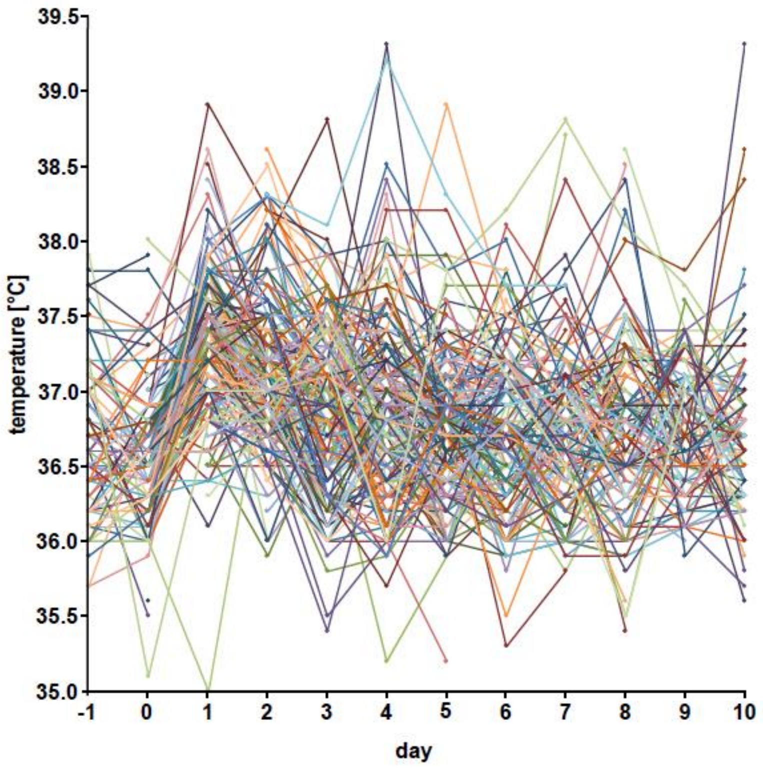

Figure S1c. Time-course of body temperature patients, who underwent pulmonary resection for the treatment of lung cancer. Data from individual patients are symbolized by different colors, and data points belonging to the same individual are connected by lines.

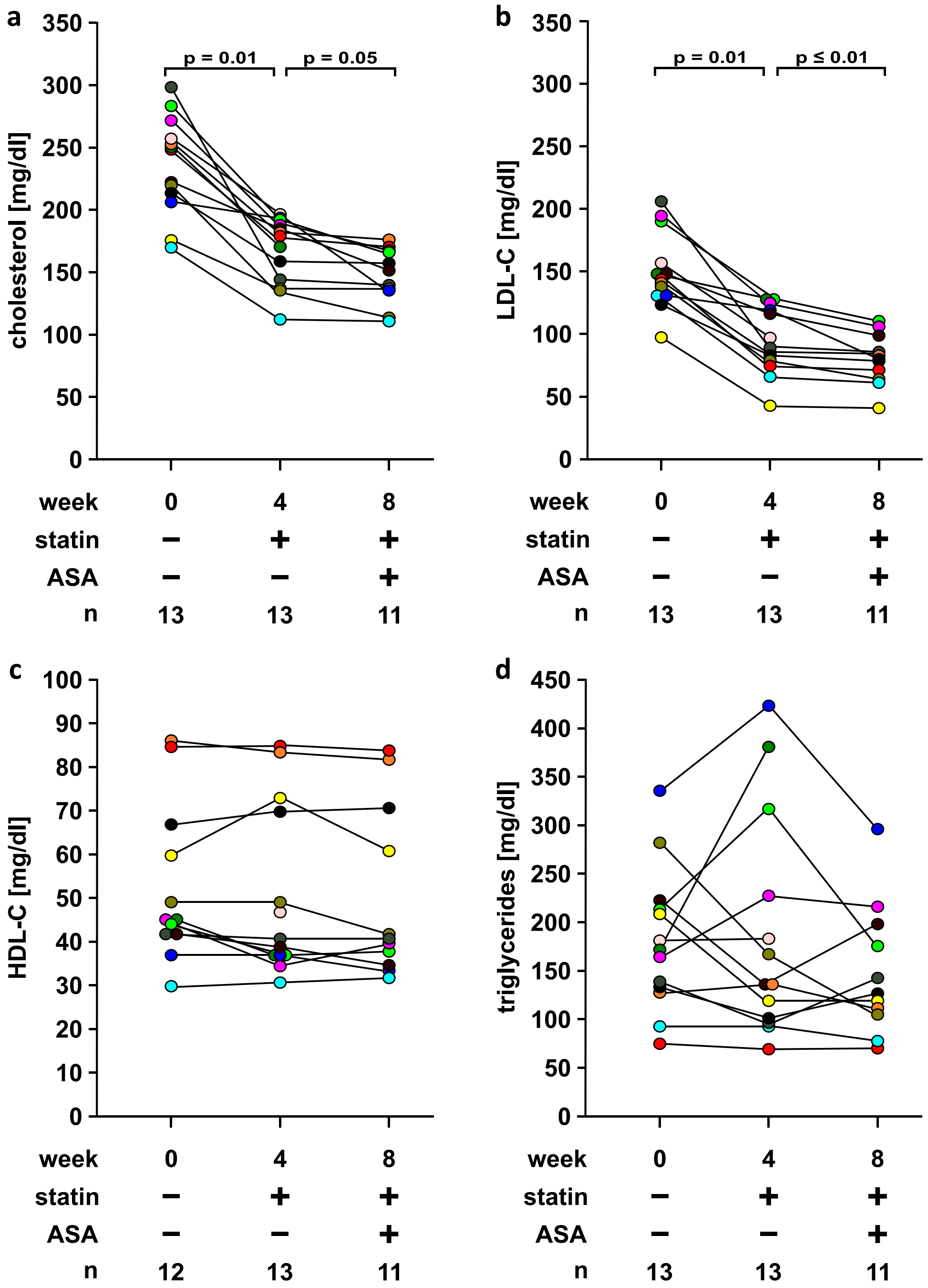

Figure S2. Serum lipids in patients suffering from mild coronary heart disease, who were investigated before treatment, after four weeks of treatment with atorvastatin or another four weeks of combined treatment with atorvastatin and acetyl salicylic acid (ASA). Serum cholesterol (**a**), low density lipoprotein cholesterol (LDL-C, **b**), high density lipoprotein cholesterol (HDL-C, **c**), data from individual patients are symbolized by different colors, and data points belonging to the same individual are connected by lines; Friedman test followed by Wilcoxon signed-rank test.

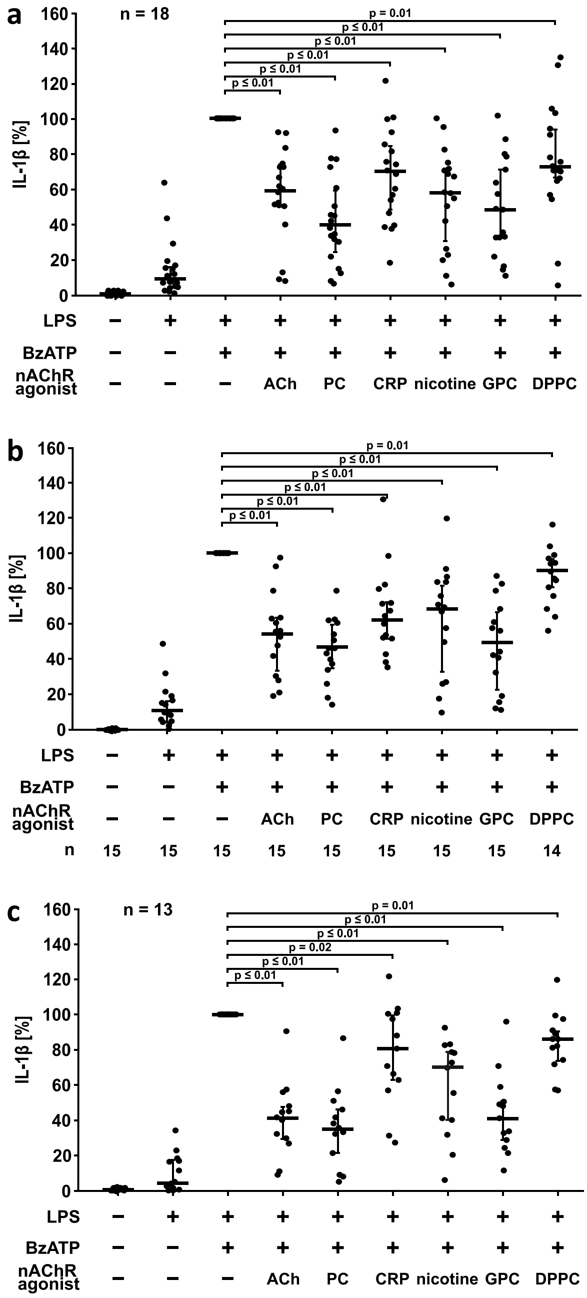

Figure S3. Cholinergic control of the release of interleukin-1β (IL-1β) by monocytes isolated from the blood of patients suffering from low-grade coronary heart disease. IL-1β was measured in cell culture supernatants of unstimulated cells, cells primed with lipopolysaccharide (LPS, 5 ng/mL, 3 h) or LPS-primed cells stimulated with 2′(3′)-O-(4-benzoylbenzoyl)adenosine 5′-triphosphate (BzATP, 100 μM, 30 min) in the presence or absence of acetylcholine (ACh, 100 μm), phosphocholine (PC, 200 μM), C-reactive protein (CRP, 5 μg/mL), nicotine (100 μM), glycero-phosphocholine (GPC, 100 μM) or dipalmitoyl phosphatidylcholine (DPPC, 100 μM). Data are presented as individual data points; bar represents median; whiskers encompass the 25th to 75th percentile. Experimental groups were compared by the Friedman test followed by the Wilcoxon signed rank test. Patient blood was drawn before lipid-lowering medication (**a**), after four weeks of statin intake (**b**) and after another four weeks of combined treatment with statin and acetylsalicylic acid (ASA) (**c**). Some of the data are also shown in Figures 8 and 9.

Suppl. Fig. 4

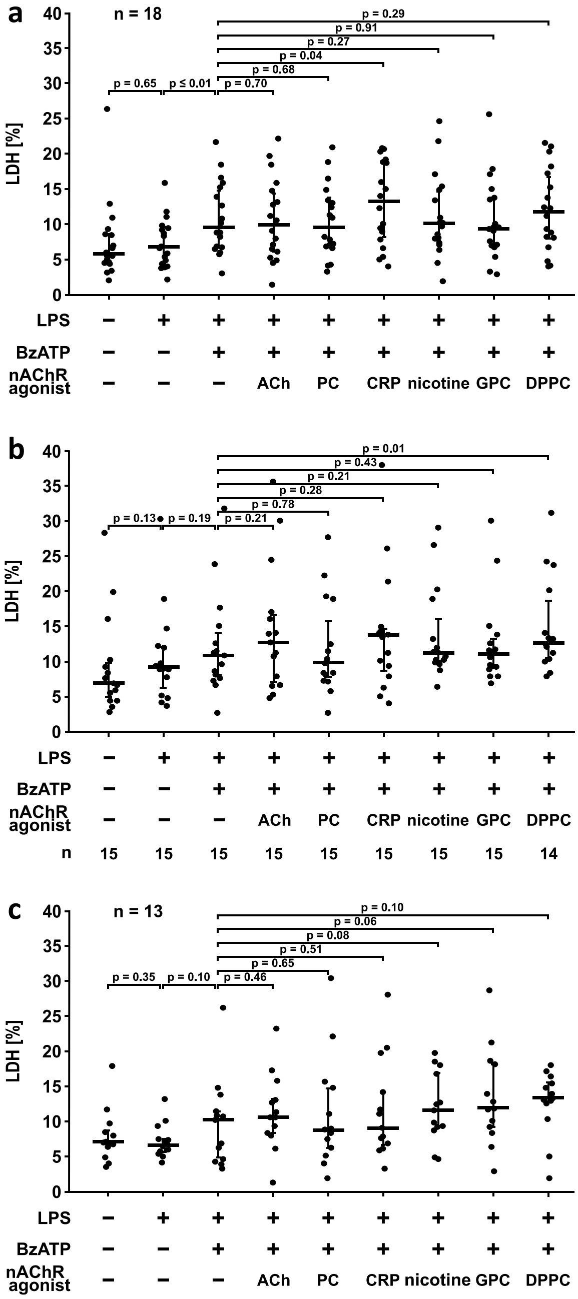

Figure S4. Release of lactate dehydrogenase (LDH) by monocytes isolated from the blood of patients suffering from low-grade coronary heart disease. LDH was measured in cell culture supernatants of unstimulated cells, cells primed with lipopolysaccharide (LPS, 5 ng/mL, 3 h) or LPS-primed cells stimulated with 2′(3′)-O-(4-benzoylbenzoyl)adenosine 5′-triphosphate (BzATP, 100 µM, 30 min) in the presence or absence of acetylcholine (ACh, 100 µm), phosphocholine (PC, 200 µM), C-reactive protein (CRP, 5 µg/mL), nicotine (100 µM), glycero-phosphocholine (GPC, 100 µM) or dipalmitoyl phosphatidylcholine (DPPC, 100 µM). Data are presented as individual data points; bar represents median; whiskers encompass the 25th to 75th percentile. Experimental groups were compared by the Friedman test followed by the Wilcoxon signed rank test. Patient blood was drawn before lipid-lowering medication (**a**), after four weeks of statin intake (**b**) and after another four weeks of combined treatment with statin and acetylsalicylic acid (ASA) (**c**).

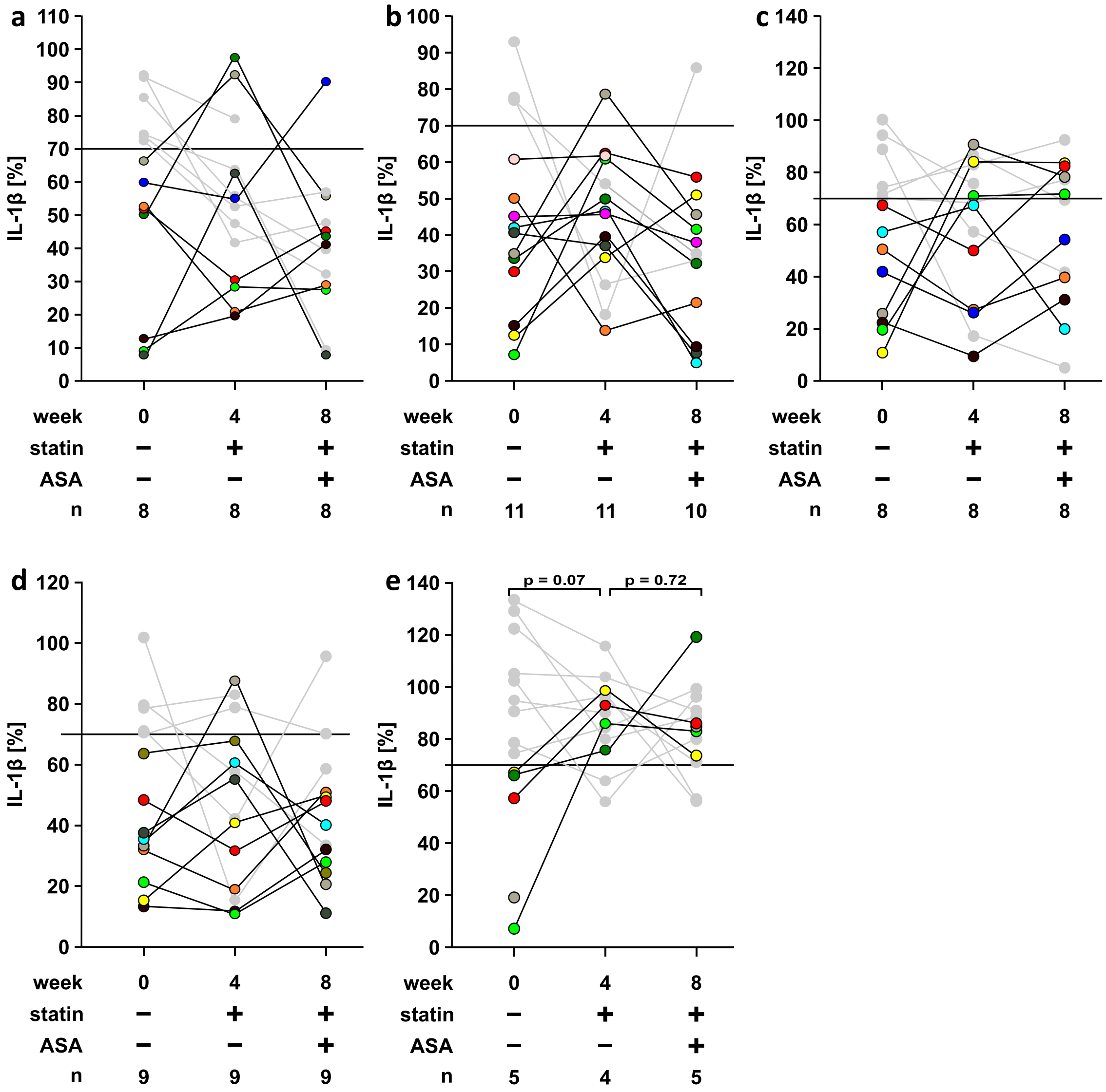

Figure S5. Inhibition of the release of monocytic interleukin-1β (IL-1β) by nicotinic agonists acetylcholine (**a**), phosphocholine (**b**), nicotine (**c**), L-α-glycerophosphocholine (**d**), or dipalmitoyl phosphatidylcholine (**e**). Patient Blood was drawn from patients suffering from low-grade coronary heart disease before lipid-lowering medication, after four weeks of statin intake and after another four weeks of combined treatment with statin and acetylsalicylic acid (ASA). IL-1β was measured in cell culture supernatants of monocytes primed with lipopolysaccharide (LPS, 5 ng/mL, 3 h) and stimulated with 2′(3′)-O-(4-benzoylbenzoyl)adenosine 5′-triphosphate (BzATP, 100 µM, 30 min) in the presence or absence of C-reactive protein (CRP, 5 µg/mL). IL-1β values obtained upon stimulation with BzATP were set to 100% and the values obtained in the presence of cholinergic agonists were calculated accordingly. Data from patients, who did not show an inhibition of IL-1β release below 70%, are depicted in grey and were not included in the statistical analysis. Data are presented as individual data points; data from individual patients are symbolized by different colors; data points belonging to the same individual are connected by lines; bar represents median; whiskers encompass the 25th to 75th percentile. Experimental groups were compared by Friedman test followed by Wilcoxon signed rank test.

Table S1. Levels of preoperative CRP (median) in patients grouped according to their respective tumor stages according to the seventh UICC guidelines.

**Tumor Stage**	**Preoperative CRP (mg/L) (*n*)**
Ia	2.75 (63)
Ib	5.09 (42)
IIa	11.21 (33)
IIb	14.34 (32)
III	10.61 (39)
IV	16.30 (7)

Table S2. Subgroup analysis regarding smoking behavior (smoker, *n* = 54; ex-smoker, *n* = 48; unknown, *n* = 115).

Day	Postopertive CRP, Smoker	Postoperative CRP, ex-Smoker	Postoperative CRP, Unknown
0	0.841 (*n* = 43, *p* ≤ 0.001)	0.897 (*n* = 38, *p* ≤ 0.001)	0.857 (*n* = 99, *p* ≤ 0.001)
1	0.325 (*n* = 52, *p* = 0.019)	0.469 (*n* = 46, *p* ≤ 0.001)	0.628 (*n* = 110, *p* ≤ 0.001)
2	0.132 (*n* = 24)	0.556 (*n* = 18, *p* = 0.017)	0.523 (*n* = 55, *p* ≤ 0.001)
3	0.188 (*n* = 27)	0.468 (*n* = 23, *p* = 0.024)	0.643 (*n* = 65, *p* ≤ 0.001)
4	0.224 (*n* = 35)	0.382 (*n* = 25)	0.456 (*n* = 66, *p* ≤ 0.001)
5	0.644 (*n* = 22, *p* ≤ 0.001)	-0.205 (*n* = 18)	0.433(*n* = 56, *p* ≤ 0.001)
6	0.710 (*n* = 20, *p* ≤ 0.001)	-0.109 (*n* = 16)	0.312 (*n* = 50, *p* = 0.028)
7	0.116 (*n* = 20)	0.358 (*n* = 20)	0.346 (*n* = 51, *p* = 0.013)
8	0.338 (*n* = 16)	0.167 (*n* = 9)	0.259 (*n* = 42)
9	-0.055 (*n* = 13)	0.055 (*n* = 11)	0.431 (*n* = 35, *p* = 0.010)
10	-0.077 (*n* = 14)	0.317 (*n* = 9)	0.538 (*n* = 30, *p* = 002)
**day**	**leuko, smoker**	**leuko, ex-smoker**	**leuko, unknown**
0	0.217 (*n* = 49)	0.133 (*n* = 46)	-0.099 (*n* = 110)
1	0.097 (*n* = 52)	0.163 (*n* = 46)	0.113 (*n* = 114)
2	-0.099 (*n* = 24)	0.612 (*n* = 18, *p* = 0.007)	0.103 (*n* = 56)
3	0.182 (*n* = 28)	0.195 (*n* = 22)	0.246 (*n* = 65, *p* = 0.049)
4	0.231 (*n* = 35)	0.066 (*n* = 25)	0.200 (*n* = 66)
5	0.326 (*n* = 23)	0.235 (*n* = 18)	0.212 (*n* = 56)
6	0.460 (*n* = 20, *p* = 0.041)	-0.059 (*n* = 17)	0.006 (*n* = 50)
7	0.162 (*n* = 20)	-0.098 (*n* = 20)	0.207 (*n* = 50)
8	0.421 (*n* = 16)	-0.286 (*n* = 8)	0.066 (*n* = 42)
9	0.390 (*n* = 13)	-0.118 (*n* = 11)	0.169 (*n* = 36)
10	0.508 (*n* = 14)	0.517 (*n* = 9)	0.078 (*n* = 30)
**day**	**temp, smoker**	**temp, ex-smoker**	**temp, unknown**
0	0.138 (*n* = 38)	-0.001 (*n* = 34)	0.161 (*n* = 78)
1	-0.204 (*n* = 42)	-0.257 (*n* = 36)	-0.022 (*n* = 86)
2	-0.076 (*n* = 49)	-0.049 (*n* = 41)	0.028 (*n* = 92)
3	0.031 (*n* = 46)	0.147 (*n* = 44)	-0.061 (*n* = 97)
4	-0.085 (*n* = 49)	0.011 (*n* = 41)	0.208 (*n* = 95, *p* = 0.043)
5	0.019 (*n* = 51)	0.094 (*n* = 39)	-0.026 (*n* = 92)
6	0.065 (*n* = 50)	-0.302 (*n* = 32)	0.117 (*n* = 86)
7	-0.135 (*n* = 48)	-0.020 (*n* = 34)	0.090 (*n* = 75)
8	-0.056 (*n* = 37)	0.082 (*n* = 28)	0.018 (*n* = 64)
9	-0.007 (*n* = 33)	0.318 (*n* = 23)	0.035 (*n* = 57)
10	0.227 (*n* = 26)	0.450 (*n* = 18)	-0.031 (*n* = 53)

Linear regression analysis of preoperative CRP values versus postoperative CRP, leukocyte number (leuko) and body temperature (temp). Smoker, ex-smoker and patients with unknown smoking behavior are compared. The correlation coefficients (r), as well as *n*-numbers and *p*-values (≤ 0.05) are given.

Table S3. Subgroup analysis regarding surgical access (VATS, $n = 152$; VATS→TT, $n = 22$; TT, $n = 43$)

Day	Postoperative CRP, VATS	Postoperative CRP, VATS→TT	Postoperative CRP, TT
0	0.826 ($n = 120$, $p \leq 0.001$)	0.923 ($n = 20$, $p \leq 0.001$)	0.895 ($n = 40$, $p \leq 0.001$)
1	0.488 ($n = 144$, $p \leq 0.001$)	0.591 ($n = 22$, $p = 0.004$)	0.276 ($n = 42$)
2	0.536 ($n = 53$, $p \leq 0.001$)	0.357 ($n = 15$)	0.175 ($n = 29$)
3	0.495 ($n = 77$, $p \leq 0.001$)	0.150 ($n = 15$)	0.405 ($n = 23$)
4	0.412 ($n = 80$, $p \leq 0.001$)	-0.022 ($n = 18$)	0.505 ($n = 28$, $p = 0.006$)
5	0.414 ($n = 59$, $p \leq 0.001$)	0.209 ($n = 13$)	0.127 ($n = 24$)
6	0.310 ($n = 60$, $p = 0.016$)	0.350 ($n = 9$)	0.397 ($n = 17$)
7	0.304 ($n = 59$, $p = 0.019$)	-0.049 ($n = 13$)	0.609 ($n = 19$, $p = 0.006$)
8	0.238 ($n = 47$)	0.486 ($n = 6$)	0.732 ($n = 14$, $p = 0.003$)
9	0.328 ($n = 42$, $p = 0.034$)	-0.371 ($n = 6$)	0.527 ($n = 11$)
10	0.377 ($n = 36$, $p = 0.023$)	-0.143 ($n = 6$)	0.200 ($n = 11$)
**day**	**leuko, VATS**	**leuko, VATS→TT**	**leuko, TT**
0	0.024 ($n = 140$)	-0.032 ($n = 22$)	-0.075 ($n = 43$)
1	0.086 ($n = 147$)	-0.119 ($n = 22$)	0.056 ($n = 43$)
2	0.149 ($n = 53$)	-0.050 ($n = 15$)	0.201 ($n = 30$)
3	0.211 ($n = 77$)	-0.202 ($n = 15$)	0.309 ($n = 23$)
4	0.191 ($n = 80$)	0.134 ($n = 18$)	0.464 ($n = 28$, $p = 0.013$)
5	0.281 ($n = 60$, $p = 0.030$)	0.259 ($n = 13$)	0.003 ($n = 24$)
6	0.039 ($n = 60$)	0.301 ($n = 9$)	0.468 ($n = 18$)
7	0.057 ($n = 58$)	0.105 ($n = 13$)	0.294 ($n = 19$)
8	0.166 ($n = 47$)	0.029 ($n = 6$)	0.604 ($n = 13$, $p = 0.029$)
9	0.207 ($n = 43$)	-0.714 ($n = 6$)	0.182 ($n = 11$)
10	0.228 ($n = 36$)	0.086 ($n = 6$)	0.009 ($n = 11$)
**day**	**temp, VATS**	**temp, VATS→TT**	**temp, TT**
0	0.152 ($n = 105$)	-0.098 ($n = 15$)	-0.011 ($n = 30$)
1	-0.108 ($n = 121$)	-0.456 ($n = 11$)	-0.091 ($n = 32$)
2	0.032($n = 131$)	-0.199 ($n = 13$)	-0.297 ($n = 38$)
3	0.085($n = 131$)	-0.173 ($n = 17$)	-0.191 ($n = 39$)
4	0.056($n = 132$)	-0.112 ($n = 16$)	0.204 ($n = 37$)
5	-0.034($n = 125$)	-0.331 ($n = 19$)	0.231 ($n = 38$)
6	0.106($n = 111$)	-0.485 ($n = 19$, $p = 0.035$)	0.067 ($n = 38$)
7	0.158($n = 103$)	-0.675 ($n = 18$, $p = 0.002$)	-0.071 ($n = 36$)
8	-0.047($n = 80$)	-0.096 ($n = 18$)	0.121 ($n = 31$)
9	0.089($n = 73$)	-0.172 ($n = 13$)	0.277 ($n = 27$)
10	0.172($n = 65$)	-0.018 ($n = 12$)	0.120 ($n = 20$)

Linear regression analysis of preoperative C-reactive protein (CRP) values versus postoperative CRP, leukocyte number (leuko) and body temperature (temp). Video-assisted thoracoscopic surgery (VATS), conversion from VATS to thoracotomy (VATS→TT) and thoracotomy (TT) are compared. The correlation coefficients (r), as well as n-numbers and p-values (≤ 0.05) are given.

Table S4. Subgroup analysis regarding the extent of pulmonary resection (seg, *n* = 15; lob, *n* = 195; pneu, *n* = 7)

Day	Postoperative CRP, Seg	Postoperative CRP, Lob	Postoperative CRP, Pneu
0	0.767 (*n* = 13, *p* = 0.002)	0.864 (*n* = 160, *p* ≤ 0.001)	0.750 (*n* = 7)
1	0.662 (*n* = 14, *p* = 0.010)	0.483 (*n* = 187, *p* ≤ 0.001)	0.500 (*n* = 7)
2	1 (*n* = 4)	0.404 (*n* = 86, *p* ≤ 0.001)	0.286 (*n* = 7)
3	0.698 (*n* = 6)	0.544 (*n* = 103, *p* ≤ 0.001)	-0.257 (*n* = 6)
4	0.5 (*n* = 5)	0.430 (*n* = 115, *p* ≤ 0.001)	0.486 (*n* = 6)
5	0.829* (*n* = 6, *p* = 0.042)	0.413 (*n* = 83, *p* ≤ 0.001)	0.429 (*n* = 7)
6	0.429 (*n* = 6)	0.337 (*n* = 77, *p* = 0.003)	0.500 (*n* = 3)
7	0.296 (*n* = 7)	0.344 (*n* = 78, *p* = 0.002)	0.771 (*n* = 6)
8	0.2 (*n* = 4)	0.306 (*n* = 60, *p* = 0.017)	0.500 (*n* = 3)
9	1 (*n* = 2)	0.249 (*n* = 55)	1.000 (*n* = 2)
10	-1 (*n* = 2)	0.258 (*n* = 47)	0.400 (*n* = 4)
**day**	**leuko, seg**	**leuko, lob**	**leuko, pneu**
0	-0.206 (*n* = 15)	0.034 (*n* = 183)	-0.214 (*n* = 7)
1	-0.173 (*n* = 14)	0.097 (*n* = 191)	-0.500 (*n* = 7)
2	0.4 (*n* = 4)	0.088 (*n* = 87)	-0.536 (*n* = 7)
3	0.395 (*n* = 6)	0.169 (*n* = 103)	-0.086 (*n* = 6)
4	-0.1 (*n* = 5)	0.163 (*n* = 115)	0.086 (*n* = 6)
5	0.257 (*n* = 6)	0.206 (*n* = 84)	0.000 (*n* = 7)
6	0.429 (*n* = 6)	0.05 (*n* = 78)	-0.500 (*n* = 3)
7	0.222 (*n* = 7)	0.131 (*n* = 77)	-0.551 (*n* = 6)
8	0.2 (*n* = 4)	0.166 (*n* = 59)	-0.500 (*n* = 3)
9	1 (*n* = 2)	0.139 (*n* = 56)	-1.000 (*n* = 2)
10	1 (*n* = 3)	0.156 (*n* = 47)	-0.800 (*n* = 4)
**day**	**temp, seg**	**temp, lob**	**temp, pneu**
0	0.549 (*n* = 10)	0.067 (*n* = 133)	-0,126 (*n* = 7)
1	-0.106 (*n* = 11)	-0.099 (*n* = 153)	(*n* = 0)
2	0.126 (*n* = 14)	-0.036 (*n* = 168)	(*n* = 0)
3	0.122 (*n* = 14)	-0.031 (*n* = 169)	-0.600 (*n* =4)
4	0.05 (*n* = 14)	0.063 (*n* = 167)	-0.200 (*n* = 4)
5	0.174 (*n* = 14)	0.002 (*n* = 163)	-0.100 (*n* = 5)
6	0.184 (*n* = 11)	0.027 (*n* = 153)	0.800 (*n* = 4)
7	-0.019 (*n* = 11)	0.031 (*n* = 141)	0.308 (*n* = 5)
8	0.049 (*n* = 8)	-0.022 (*n* = 115)	0.657 (*n* = 6)
9	0.718 (*n* = 7)	-0.01 (*n* = 102)	0.000 (*n* = 4)
10	0.947 (*n* = 5, *p* = 0.014)	-0.024 (*n* = 88)	-0.316 (*n* = 4)

Linear regression analysis of preoperative C-reactive protein CRP values versus postoperative CRP, leukocyte number (leuko) and body temperature (temp). Segmentectomy (seg), lobectomy (lob) and pneumonectomy (pneu) are compared. The correlation coefficients (r), as well as *n*-numbers and *p*-values (≤ 0.05) are given.
